# Time to Publication in Medical Education Journals: An Analysis of Publication Timelines During COVID-19 (2019–2022)

**DOI:** 10.5334/pme.1287

**Published:** 2024-10-11

**Authors:** Lauren A. Maggio, Joseph A. Costello, Kirsten R. Brown, Anthony R. Artino, Steven J. Durning, Ting Lan Ma

**Affiliations:** 1 University of Illinois Chicago, College of Medicine, Illinois, USA; 2 Center for Health Professions Education, Uniformed Services University of the Health Sciences in Bethesda, Maryland, USA; 3 George Washington University School of Medicine and Health Sciences, Washington, DC, USA; 4 Center for Health Professions Education at the Uniformed Services University of the Health Sciences in Bethesda, Maryland, USA; 5 Health Professions Education at Uniformed Services University of the Health Sciences in Bethesda, Maryland, USA

## Abstract

**Introduction:**

COVID-19 changed scholarly publishing. Yet, its impact on medical education publishing is unstudied. Because journal articles and their corresponding publication timelines can influence academic success, the field needs updated publication timelines to set evidence-based expectations for academic productivity. This study attempts to answer the following research questions: did publication timelines significantly change around the time of COVID-19 and, if so, how?

**Methods:**

We conducted a bibliometric study; our sample included articles published between January 2018, and December 2022, that appeared in the Medical Education Journals List-24 (MEJ-24). We clustered articles into three time-based groups (pre-COVID, COVID-overlap, and COVID-endemic), and two subject-based groups (about COVID-19 and not about COVID-19). We downloaded each article’s metadata from the National Library of Medicine and analyzed data using descriptive statistics, analysis of variance, and post-hoc tests to compare mean time differences across groups.

**Results:**

Overall, time to publish averaged 300.8 days (*SD* = 200.8). One-way between-groups ANOVA showed significant differences between the three time-based groups *F* (2, 7473) = 2150.7, *p* < .001. The post-hoc comparisons indicated that COVID-overlap articles took significantly longer (*n* = 1470, *M*= 539; *SD* = 210.6) as compared to pre-COVID (*n* = 1281; *M* = 302; *SD* = 172.5) and COVID-endemic articles (*n* = 4725; *M* = 226; *SD* = 136.5). Notably, COVID-endemic articles were published in significantly less time than pre-pandemic articles, *p* < .001.

**Discussion:**

Longer publication time was most pronounced for COVID-overlap articles. Publication timelines for COVID-endemic articles have shortened. Future research should explore how the shift in publication timelines has shaped medical education scholarship.

## Introduction

Journal articles are an important metric of academic productivity, and publication success is a significant consideration in tenure and promotion decisions. However, the publication process and its timeliness are influenced by factors beyond the quality of the scholarship (e.g., reviewer availability, quality, and responsiveness). Prior to the COVID-19 pandemic it took, on average, 263 days from submission of an article to a medical education journal for it to appear in PubMed [1]. This finding was based on articles published between 2008–2018. Since this publication, the COVID-19 pandemic has changed the world and consequently scholarly communication [2]. While multiple studies have investigated the impact of COVID-19 on publishing broadly [3,4] and in biomedicine more specifically [5], the pandemic’s effect on medical education publishing is unstudied.

COVID-19 was a “game changer” in medical education [6]. Yet, it is unknown how COVID-19 shaped publishing in medical education and if publication-related factors observed in other fields (e.g., publication type and coverage of COVID-19) played a role in publication timelines. As publications and their corresponding timelines are crucial determinants of academic success, it is critical for the field to have an updated publication timeline that provides insights into recent publication timelines otherwise unknown. Accurate timelines enable stakeholders to set realistic expectations for productivity for the COVID-19 time period and, as needed, to advocate for their members. Publication timelines also provide the consumers of these publications with a sense of the recency of an article’s content, which is important because findings from such articles can often influence educational policy and practice.

COVID-19 shifted the dynamics of scholarly publishing. Researchers found that on the one hand, COVID-19 “turbocharged” some scholarly publishing [5] such that the speed and volume of publication increased to provide rapid evidence to front-line healthcare workers, policy makers, and the public [3,7]. For example, one study found that early in the pandemic, medical journals halved their time to publication [8] and articles about the pandemic itself were published at faster rates and higher volumes than those not related to the pandemic [2,9,10]. On the other hand, publication of non-COVID focused research slowed [2], the productivity of certain groups (e.g., females, physician scientist trainees, caregivers, medical faculty) diminished [11,12,13,14,15], and the lack of access to classrooms and labs delayed research that was in the data collection and analysis phase.

Journal publishers and editors responded to COVID-19 in a variety of unprecedented ways. For example, to expedite publication, 20 major publishers and scholarly organizations united to establish the C19 Rapid Review initiative through which they shared peer reviews and the identities of reviewers thus streamlining the flow of manuscripts across journals [16]. In two other instances, the journal *eLife* adapted its peer review policies such that reviewers were advised not to require additional experiments or analyses in revision requests, because these requests would hinder publication speed [17], and the *Lancet* expanded their ‘fast tracking’ system for articles to expedite the sharing of ‘critical knowledge’ [5]. Recognizing the pandemic’s toll on reviewers and authors, many journals, including those in medical education, extended peer review and revision timelines [18]. Across science broadly there is some evidence as to how such initiatives impacted publication timelines [19,20], but there is no evidence specific to medical education.

At the start of the pandemic, *eLife’s* editor-in-chief espoused: “Publishing will not and should not be anybody’s first priority in the coming months” [17]. However, now that we are well beyond the pandemic’s emergency period, it is hard to ignore academia’s long history of prioritizing journal articles as a marker of success [21], which has high stakes implications for the scholarly progression of individuals and the field. At the individual level, journal articles and their related publication timelines play a role in one’s ability to be promoted, to secure grant funding, and to graduate from publication-dependent degree programs. For example, a review of promotion and tenure guidelines for faculty of medicine at 92 universities identified that 95% of guidelines mentioned peer-reviewed publications with 35 institutions (38%) specifying a required number of articles [22]. Thus, it is important for individuals and administrators to have updated publication timelines that reflect the reality of publishing during COVID-19.

Although the pandemic has ended and COVID-19 is now endemic, the potential for future pandemics or natural disasters is high, such that understanding these timelines is still an important aim. First, it will help to ensure that realistic expectations for productivity are being set and that evidence is available for advocating for researchers who may have faced a publication timeline influenced by the pandemic. An updated publication timeline also informs evidence-based decision making. Ideally, individuals make decisions using the “best available evidence” [23] with the currency of that evidence being a factor that enables the user to determine the relevance of the evidence to their current context. Thus, in this study, we explore publication timelines in medical education just before and during the COVID-19 pandemic. To guide this study, we asked: did publication timelines significantly change around COVID-19 and, if so, what were those changes, both broadly and in relation to publishing characteristics such as publication type and whether or not an article was about COVID-19.

## Methods

We conducted a bibliometric study that replicated and expanded upon a prior study on publication timelines in medical education [1]. That prior work drew heavily upon an earlier study that had investigated this topic broadly across scientific disciplines [24]. Where deviations were made from the original study [1] we make note and provide a rationale.

### Sample

We included articles that appeared in the Medical Education Journals List (MEJ-24) and that are indexed by PubMed. The MEJ-24 is a seed set of journals that was derived using bibliometric methods to represent the field of medical education [25]. The MEJ-24 includes 24 journals and represents an expansion from the previous study, which considered 14 journals [1].

**Figure 1:**
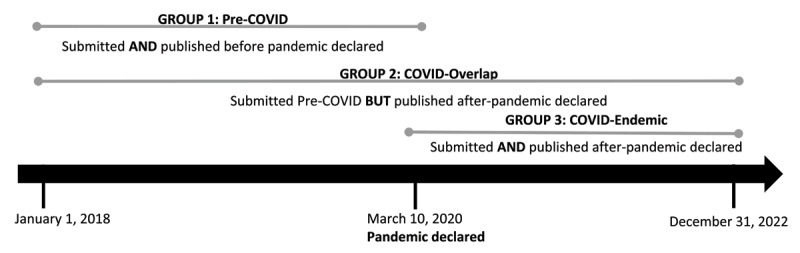
Pre-COVID, COVID-overlap, and COVID-endemic periods.

Our sample included articles published in any one of the MEJ-24 journals between January 1, 2018 and December 31, 2022. To investigate the potential differences in publication times before and after the COVID-19 pandemic, we clustered articles into the following three groups (See Figure 1). The creation of these three groups deviates from the earlier study, which considered all articles across the study period as the same.

Group 1 (G1; pre-COVID) included articles submitted between January 1, 2018–March 10, 2020 and published prior to March 10, 2020. We selected March 10, 2020 as a cutoff date for publication as the World Health Organization declared a global pandemic on this date [26]. This group served as our control, since the COVID-19 pandemic should not have affected these articles’ publication time. We conceptualized Group 2 (G2; COVID-overlap) as an overlap period that contains articles submitted before the COVID outbreak, but not published until after the COVID outbreak. G2 articles were submitted between January 1, 2018 and March 10, 2020 and published after March 10, 2020. Group 3 (G3; COVID-endemic) included articles submitted after March 10, 2020 and published by December 31, 2022. We descriptively summarize the number of days in publication timeline for these three periods and compare the mean-level differences for statistical significance.

### Data Collection

To collect our data, we referenced *The History of Publication Delays* [27] an analysis of publication delays up to 2015 utilizing the entire PubMed database. We used the original code created by Himmelstein [28] that is deposited on his Github account with only minor modifications. The modifications included an update for a depreciated function within a Python package and minor adjustments to account for a smaller dataset. On October 10, 2023 JC queried the eSearch e-utility, an API interface, using the updated code to download summary metadata from the National Library of Medicine (NLM) for all included articles [29]. Metadata included: Journal name, PubMed ID (PMID), NLM Journal Id, Article Type, Citation, Abstract, References, and Article History (Received Date, Accepted Date, and PubMed Publication Date). To be included in the analysis, it was necessary that an article was received by a publisher in the MEJ-24 on or after January 1, 2018 and published by the same journal before December 31, 2022. All metadata was merged as a table and exported to a CSV file.

We relied on the NLM’s indexing to determine an article’s publication type (e.g., review, letter, clinical trial) and if it was about COVID-19. To determine publication types, we used the publication types as defined and applied by the NLM. To characterize articles about COVID, we utilized NLM LitCOVID, which is a comprehensive search of PubMed for articles about COVID [30,31]. We made the judgment that an article was likely not peer reviewed, by identifying the articles with the following NLM publication types: Comment, Editorial, Letter, News, Published Erratum, or Retracted Publication. All other publication types were considered to be peer reviewed.

We also needed to account for articles where the publisher submitted an accepted date that was prior to a received date (*n* = 18) or a published date that was prior to an accepted date (*n* = 5).

### Analysis

Data were analyzed using SPSS version 28 [32]. To analyze the data, we first reported descriptive statistics. To compare publication timeline differences for articles that were published pre-COVID and after-COVID (the latter is further separated into articles submitted pre-COVID and after-COVID), we conducted analyses of variance (ANOVA) and compared mean differences across groups. Upon the significance level of an ANOVA Omnibus *F* test (*p* < .05), a follow-up LSD post hoc pairwise comparison was conducted to determine where the significance was between any one of the three group comparisons. We also conducted independent-samples t-tests to compare mean differences in publication timeline between COVID vs. non-COVID related articles. Timeline differences in articles of different publication types were also explored. Cohen’s *d* was reported upon significance to determine the effect sizes.

## Results

During the study period 16,544 articles were published in MEJ-24 journals (See Zenodo for dataset [33]). Of these journals, 15 supplied partial or complete publication timeline data representing 8,501 (51.4%) articles (See Online Appendix A). For the purposes of our research question, we focused on articles submitted after January 1, 2018 and published prior to December 31, 2022 with full timeline data. This represented 7,614 articles from 15 journals of which 23 articles included flawed metadata (e.g. the received date was after the acceptance date) so were removed resulting in 7,591 (45.9%) articles included for analysis.

The time to publish was on average 188.1 days (*SD* = 115.3). Time from submission to acceptance was on average 149.9 days (*SD* = 106.5). Processing time, which is the time between acceptance and appearing in PubMed, was on average 38.2 days (*SD* = 46.1).

One-way between-group ANOVA results showed the publication time was significantly different among Group 1 (pre-COVID), Group 2 (COVID-overlap), and Group 3 (COVID-endemic), *F* (2, 7588) = 237.66, *p* < .001. Significant differences were also found for acceptance time, *F* (2, 7588) = 177.10, *p* < .001, and process time, *F* (2, 7588) = 79.58, *p* < .001. These results indicated publication timeline was significantly different during pre-COVID (G1), COVID-endemic (G3), and when there was overlap during COVID (G2, i.e., submitted pre-COVID but published after-COVID).

The post-hoc comparisons using the Tukey HSD test indicated that articles of Group 2 (COVID-overlap, *n* = 761, *M* = 262.8; *SD* = 134.4) took significantly longer time in publication as compared to articles in Group 1 (both submitted and published before COVID, *n* = 2004; *M* = 200.7; *SD* = 118), and those in Group 3 (both submitted and published COVID-endemic, *n* = 4826; *M* = 171.1; *SD* = 105.2). Significant results with the same trend were found for acceptance time, and for processing time (see Table 1 for *M*s and *SD*s).

**Table 1:** Publication timelines for articles published in the MEJ-24 between 2018–2022 with available publication metadata.


		TIME TO PUBLICATION			TIME TO ACCEPTANCE			PROCESSING TIME		
		
	N	MEDIAN	MEAN	SD	MEDIAN	MEAN	SD	MEDIAN	MEAN	SD

Overall articles	7591	165.0	188.1	115.3	129.0	149.9	106.5	23.0	38.2	46.1

***Time Frame***										

G1-Pre-COVID	2004	181.0	200.7	118.0	133.0	154.7	109.0	27.0	46.0	53.3

G2-COVID-overlap	761	246.0	262.8	134.4	197.0	213.6	129.4	24.0	49.1	57.3

G3-COVID-endemic	4826	151.0	171.1	105.2	119.0	137.9	97.3	23.0	33.3	39.7

***COVID-Related***										

Non-COVID-related	6736	169.0	192.1	116.7	133.0	153.5	107.8	24.0	38.5	46.3

COVID-related	855	141.0	156.8	98.5	99.0	121.1	90.6	22.0	35.7	44.3

G3-Non-COVID-related	3975	152.0	174.2	106.5	123.0	141.4	98.4	23.0	32.8	38.7

G3-COVID-related	851	141.0	156.6	97.6	100.0	121.1	90.1	22.0	35.5	44.0

***Peer-Reviewed***										

Peer-reviewed	7139	172.0	196.4	112.7	135.0	157.5	104.4	24.0	38.9	46.2

Non-peer-reviewed	452	33.0	57.0	66.8	9.0	30.2	53.5	15.0	26.8	43.3


G1 – Group 1, G2 – Group 2, G3 – Group 3. Note. In our previous analysis, we hypothesized that the first 6 months after the COVID-19 outbreak would be particularly challenging for authors with parenting responsibilities, as most K-12 schools and preschools were closed. To explore this further, we closely examined two subgroups: 3a) articles published during March–June 2021 (0–3 months post-outbreak), M = 213, SD = 132.28, and 3b) articles published during June-September 2021 (3–6 months post-outbreak), M = 208, SD = 123.46. Findings showed that the first subgroup has a slightly longer publication timeline than the pre-covid stage, although it was still shorter than that of Group 2. Group 3b showed no statistically significant difference in timeline compared to the pre-COVID stage. Overall, the timelines of these micro stages still went down compared to Group 2.

The results showed that articles that were submitted when COVID was endemic (Group 3) were published significantly faster than those that were submitted and published before the pandemic (Group 1), *p* < .001. The same significant results were found between these two groups for acceptance time and process time (see Table 1 for *M*s and *SD*s). Thus, these results indicate that the publication timelines were delayed specifically for those articles submitted prior to/close to the pandemic outbreak, but not for those that were submitted once COVID became endemic.

### COVID-Related Articles

When compared to non-COVID articles in general (across Groups 1–3), COVID-related articles were published significantly faster, *t* (7589) = –8.46, *p* < .001, Cohen’s *d* = 0.31. When looking into specific period, for example, among Group 3 (COVID-endemic), articles that were COVID-related had significantly shorter publication timeline (*n* = 851; *M* = 156.6; *SD* = 97.6) compared to those that were non-COVID related (*n* = 3975; *M* = 174.2; *SD* = 106.5), *t* (4824) = –4.43, *p* < .001, mean difference = –17.59, 95% *CI*: –25.36 to –9.82, Cohen’s *d* = 0.17. The same trend with significant results were also found for acceptance time and process time (see Table 1 for *M*s and *SD*s). However, non-COVID-related articles during the pandemic were still published significantly faster than those that were pre-pandemic, *t* (5977) = –8.82, *p* < .001.

### Publication Types

The NLM indexed articles as a variety of publication types (Table 2). Commentaries had the shortest publication time of 36.5 days (*n* = 271; *SD* = 37.1; *median* = 25) followed by editorials, which took on average 72.6 days (*n* = 107; *SD* = 81.3; *median* = 50). Case reports, guidelines, news, and published erratum also had relatively short timelines, but there were fewer articles representing these publication types compared to the other types. Among all types, peer-reviewed articles took significantly longer time to publish, *M* = 196.4; *SD* = 112.7, *t* (627) = 40.84, *p* < .001, mean difference = 139.38, 95% *CI*: 132.67–146.08, Cohen’s *d* = 1.26, compared to those that were not peer-reviewed (*M* = 57; *SD* = 66.8). Significant results with the same trend were found for acceptance time, and for processing time (see Tables 1 and 2 for *M*s and *SD*s).

**Table 2:** Publication timelines by publication types.


PUBLICATION TYPES*		PUBLICATION TIME			ACCEPTANCE TIME			PROCESSING TIME		
		
	N	MEDIAN	MEAN	SD	MEDIAN	MEAN	SD	MEDIAN	MEAN	SD

Case Reports	1	88.0	88.0	–	50.0	50.0	–	38.0	38.0	–

Clinical Trial	3	272.0	278.3	127.6	252.0	252.0	146.0	20.0	26.3	19.3

Comment	271	25.0	36.5	37.1	9.0	19.6	32.8	10.0	16.9	19.1

Comparative Study	1	171.0	171.0	–	156.0	156.0	–	15.0	15.0	–

Editorial	107	50.0	72.6	81.3	6.0	16.9	30.6	34.0	55.6	75.7

Guideline	1	1.0	1.0	–	0.0	0.0	–	1.0	1.0	–

Historical Article	11	140.0	164.5	91.5	99.0	136.7	98.4	15.0	27.8	34.0

Journal Article	7299	169.0	192.4	114.1	133.0	154.2	105.6	24.0	38.2	45.3

Letter	169	46.0	74.3	74.3	22.0	55.8	72.9	14.0	18.5	16.6

Meta-Analysis	36	160.0	177.2	87.6	136.5	158.1	87.1	15.5	19.1	14.7

Multicenter Studies	116	173.5	188.5	85.6	146.0	156.4	84.4	23.0	32.1	31.7

News	2	93.5	93.5	21.9	36.5	36.5	12.0	57.0	57.0	9.9

Published Erratum	15	22.0	22.1	14.7	0.0	1.2	2.2	21.0	20.9	15.2

Randomized Controlled Trial	274	203.0	227.3	123.0	171.0	198.7	116.4	18.0	28.6	32.3

Retracted Publication	3	98.0	77.0	51.8	0.0	26.7	46.2	18.0	50.3	56.0

Review	395	165.0	190.3	115.7	127.0	146.3	98.6	24.0	44.0	62.5

Peer-reviewed	7139	172.0	196.4	112.7	135.0	157.5	104.4	24.0	38.9	46.2


*Articles may be assigned more than one publication type.

### Publications by Journal

Publication timelines for each included journal are reported in Table 3. There is variation in terms of publication timeline for each specific journal. As a robustness check for our main ANOVA analyses, we removed the journal with the longest publication time, *Anatomical Science Education*, and the results remain the same.

**Table 3:** Publication timelines by journal.


JOURNALS		PUBLICATION TIME			ACCEPTANCE TIME			PROCESSING TIME		
		
	N	MEDIAN	MEAN	SD	MEDIAN	MEAN	SD	MEDIAN	MEAN	SD

*Advances in Health Sciences Education*	310	228.5	251.3	136.5	210.0	227.4	132.7	19.0	23.9	16.8

*Advances in Medical Education And Practice*	649	115.0	124.4	72.7	79.0	80.2	54.4	33.0	44.1	36.3

*Anatomical Sciences Education*	347	225.0	226.9	129.6	215.0	215.7	130.3	6.0	11.2	19.3

*BMC Medical Education*	2524	193.5	216.3	108.2	173.5	195.9	106.9	16.0	20.4	15.7

*BMJ Simulation & Technology Enhanced Learning*	108	179.0	240.5	170.7	92.0	93.1	67.5	29.0	147.4	163.0

*Clinical Teacher*	239	165.0	180.7	118.3	118.0	144.4	122.7	32.0	36.3	18.5

*GMS Journal for Medical Education*	370	327.5	312.4	133.2	190.5	187.1	106.2	119.0	125.3	52.9

*International Journal of Medical Education*	186	195.0	208.6	93.4	173.5	185.5	92.6	21.5	23.1	10.9

*Journal of Continuing Education in the Health Professions*	3	316.0	279.7	131.3	258.0	213.7	128.4	65.0	66.0	8.5

*Journal of Educational Evaluation for Health* *Professions*	183	41.0	52.6	56.1	31.0	40.0	47.1	3.0	12.6	30.6

*Journal of Graduate Medical Education*	301	289.0	286.2	58.1	173.0	174.7	53.4	111.0	111.5	26.7

*Journal of Medical Education and Curricular* *Development*	308	112.0	129.7	84.7	48.0	59.6	63.8	55.0	70.2	55.3

*Journal of Surgical Education*	1098	135.5	145.9	66.2	101.0	109.8	61.2	29.0	36.1	22.5

*Medical Education*	852	125.0	120.5	85.0	97.0	94.7	68.5	9.0	25.8	35.4

*Perspectives on Medical Education*	113	196.0	190.8	111.0	154.0	154.7	105.4	32.0	36.1	17.4


## Discussion

The pandemic phase of COVID-19 is over; yet this short-lived period had a profound impact on science and academic publishing [34]. Our findings suggest that this effect was also felt by those publishing in medical education, but these effects can be considered both positive and negative. For example, articles submitted during the COVID-overlap period took almost a year and a half to be published, which may have had consequences for individuals and the currency of the field’s knowledge base. However, in the COVID-endemic period, we observed that even with the increased number of manuscripts submitted to and published by medical education journals, the timeline for publication was significantly shorter, suggesting that there may be efficiencies worth exploring and possibly retaining into the future.

This study confirms findings from the broader scientific literature (e.g., biomedicine [35]): we found that the publication timeline for COVID-19 topics was quicker than publication timelines for non-COVID topics. This effect may have been the result of the various mechanisms that journals implemented. For example, it was found that the peer review process could be made faster because editors collaborated with editors at other journals [36] or removed hurdles such as requests for additional data [17]. While these mechanisms were introduced in a time of crisis and for a specific topic, it is important for editors and publishers to consider which, if any, should be retained in the future. Additionally, researchers should study the costs that these mechanisms might bring to the community (e.g., stress on peer reviewer effort, lessening of article quality) and the degree to which they are tolerable.

As noted, the time to publication for COVID-endemic articles was shorter than those submitted before and at the pandemic’s beginning. This finding was surprising based on research from other fields [2,4] and our personal experiences as authors who had submitted manuscripts to HPE journals across the studied timeframe. The cause of this uptick in publication speed requires additional research to be fully understood, especially regarding whether this increased pace can be maintained. However, drawing on our experience as authors, reviewers, and editors, we speculate that the faster pace of publishing during the COVID-endemic period may be an artifact of editors and publishers becoming accustomed to and integrating lessons learned from the earlier periods of the pandemic when they were challenged to deal with a proliferation of manuscripts previously unexperienced. In other words, editors and their teams became accustomed to the high volume of manuscripts and were able to employ strategies that translated into quicker turnaround times. It is also possible that processes, such as the Fast Track option offered by *Advances in Health Sciences Education* [37] or the similar TakeTwo publication type at *Perspectives on Medical Education* [38], which take advantage of existing peer reviews, may have contributed to this increased pace. Nonetheless, more research is needed to corroborate these speculations.

This study extends the existing literature by providing updated publication timelines and evidence-based expectations for academic productivity during the COVID-19 time period. In examining the COVID-pandemic effect, articles submitted between January 1, 2018 and March 10, 2020, and those published after March 10, 2020, took substantially longer to move through the publication process. So, while many universities granted “the gift of time” by automatically extending faculty tenure clocks during the pandemic [39], tenure and promotion committees must consider that many articles were impacted, including not just those that were submitted during the pandemic phase, but also articles under review or in revisions prior to COVID that were significantly affected. Thus, faculty hired between 2018 and 2020 might be expected to have less research productivity (at least in terms of published articles) during this period. Faculty seeking tenure and promotion could use these findings in a statement about how COVID shaped their academic productivity during the COVID-19 period to explain potential gaps in publishing productivity. We observed that the article type (e.g., commentary, review article) should also be considered when assessing productivity, as time to publication can vary considerably between publication types. Lastly, authors and promotion committees should consider that this slowdown may also impact the quantity of citations an author’s manuscript may accrue.

Previous research found that medical education articles took an average of 263 days to publish between 2008 and 2018 [1]. Since March 11, 2020, articles are taking an average of 226 days from submission to appearing in PubMed. While shorter, this more recent timeline stands in contrast to the estimated 100 days it takes for the biomedical articles to be published [24], which has been criticized as a lengthy publication delay with potential negative effects on scientific progress [40]. As a field, medical education must consider what such a delay means for the timeliness of the evidence that is used to make decisions and craft educational policies.

### Limitations and Areas for Future Research

This study has limitations. First, only 15 of the MEJ-24 journals supplied publication timeline data. However, this represented a sampling frame of over half (66.6%) of the articles published in the study’s timeframe. It is possible that journals that did not report publication timeline data in their metadata represent a form of non-response bias, if they are systematically different from the journals that did report timelines in their metadata. However, in comparison to Maggio et. al’s earlier study [1], our current sample represents an increase in availability of publication metadata. Second, we relied upon NLM indexers to determine whether or not an article was about COVID. While information scientists designed and vetted the search approach, it is possible that some articles were inadvertently missed, as researchers have identified that there can be inconsistencies in indexing [41]. This study focused on the effects of the COVID pandemic on publication timelines in medical education. However, it is worth remembering that many other events occurred during this time period that also could have influenced publication timelines, including several social and environmental events like the Black Lives Matter movement, Hong Kong protests, and Australian bushfires.

The quantitative and descriptive approach used in this study leaves unanswered many questions about publication timelines in medical education. For instance, publication timelines are composed of multiple steps undertaken by a variety of individuals (e.g., authors, editors, reviewers, copy editors, typesetters); however, the data analyzed here provides limited insight into how each of these individuals contributed to the variable publication timelines observed. In terms of impact, it is unclear if and how substantive publication delays in the COVID-overlap group affected early career authors. Additionally, it is unknown whether the steps journal editors took actually alleviated the problems associated with reviewer responsiveness or promoted COVID-specific scholarship. In light of these limitations, future qualitative studies should explore the lived experiences of authors and editors to better understand the ways in which COVID may have shaped medical education publishing.

Publication timelines are of great interest to both medical education authors and editors, who have described them as “crucial” in influencing their decision of whether to submit to a journal [42]. Thus, this study provides a unique snapshot of publication timelines for the COVID-19 period across multiple journals, which gives those engaged in publishing at that time an explanation for the tempo of their publications. However, as we move beyond the COVID-19 pandemic, this study also provides those looking forward with a replicable and streamlined method for conducting their own data collection and analysis to continue to study publication timelines in the field.

## Data Accessibility Statement

Costello JA, Maggio LA, Brown KR, Artino Jr. AR, Durning S, Ma T. Revisiting the time to publication in medical education: An analysis of publication timelines between 2019–2022 [Data set]. Zenodo. Published 2023. Available at: https://doi.org/10.5281/zenodo.10433647.

## Additional File

The additional file for this article can be found as follows:

10.5334/pme.1287.s1 Appendix A. MEJ-24 Journals with Publication Timeline Data Available for Analysis.
