# Supplementary material for: Time to Publication in Medical Education Journals: An Analysis of Publication Timelines During COVID-19 (2019–2022)
Source: Perspect Med Educ. 2024 Oct 11;13(1):507–17. doi: 10.5334/pme.1287 (PMC11468245; doi:10.5334/pme.1287)
Supplement: Appendix A. — MEJ-24 Journals with Publication Timeline Data Available for Analysis. [file pme-13-1-1287-s1.pdf]

Appendix A: MEJ-24 Journals with Publication Timeline Data Available for Analysis

Journals	Articles published	Publication Timeline Data Available	Publication Timeline Data Meeting Inclusion Criteria**	Avg. time to publication (SD)	Avg. time to acceptance (SD)	Average processing time (SD)
Academic Medicine	2980					
Advances in Health Sciences Education	380	356	310	251.3 (136.48)	227.4 (132.7)	23.9 (16.8)
Advances in Medical Education And Practice	822	650	649	124.4 (72.7)	80.2 (54.4)	44.1 (36.3)
African Journal of Health Professions Education	4					
Anatomical Sciences Education	400	389	347	226.9 (129.6)	215.7 (130.3)	11.2 (19.3)
BMC Medical Education	2850	2776	2524	216.3 (108.2)	195.9 (106.9)	20.4 (15.7)
BMJ Simulation & Technology Enhanced Learning	343	333	108	240.6 (170.7)	93.1 (67.5)	147.4 (163)
*Canadian Medical Education Journal**						
Clinical Teacher	652	245	239	180.7 (118.3)	144.4 (122.7)	36.3 (18.5)
Education for Health	158					
*Focus on Health Professional Education**						
GMS Journal for Medical Education	414	414	370	312.4 (133.2)	187.1 (106.2)	125.3 (52.9)
International Journal of Medical Education	224	224	186	208.6 (93.4)	185.5 (92.6)	23.1 (10.9)
Journal of Continuing Education in the Health Professions	269	3	3	279.7 (131.3)	213.7 (128.4)	66 (8.5)
Journal of Educational Evaluation for Health Professions	192	192	183	52.6 (56.1)	40 (47.1)	12.6 (30.6)
Journal of Graduate Medical Education	926	346	301	286.2 (58.1)	174.7 (53.4)	111.5 (26.7)
Journal of Medical Education and Curricular Development	378	354	308	129.7 (84.7)	59.6 (63.8)	70.2 (55.3)
Journal of Surgical Education	1201	1190	1098	145.9 (66.2)	109.8 (61.2)	36.1 (22.5)
Medical Education	1354	913	852	120.5 (85)	94.7 (68.5)	25.8 (35.4)
Medical Education Online	405					
Medical Teacher	1556					
Perspectives on Medical Education	315	116	113	190.8 (111)	154.7 (105.4)	36.1 (17.4)
Simulation in Healthcare	411					
Teaching and Learning in Medicine	310					

Total	16544	8501	7591			

*Not indexed in PubMed

** Submitted for peer-review on or after January 1, 2018 and Published by December 31, 2022
